# WEDGE FRAGMENT VARIATIONS OF TIBIAL SHAFT FRACTURES WITH INTRAMEDULLARY NAILING

**DOI:** 10.1590/1413-785220233103e268124

**Published:** 2023-09-08

**Authors:** Mario Sergio Boff, Pedro Henrique de Oliveira Paolucci, Gabriel Machado de Oliveira, Leonardo Zanesco, Fernando Brandao Andrade-Silva, Marcos de Camargo Leonhardt, Paulo Roberto dos Reis, Jorge dos Santos Silva, Kodi Edson Kojima

**Affiliations:** 1 Universidade de Sao Paulo, Faculdade de Medicina, Hospital das Clinicas HC-FMUSP, Instituto de Ortopedia e Traumatologia IOT, Grupo de Trauma, Sao Paulo, SP, Brazil.

**Keywords:** Tibial Fractures, Pseudarthrosis, Risk factors, Prognostic Factors, Nailing, Intramedullary, Fraturas da Tíbia, Pseudoartrose, Fatores de risco, Fatores Prognósticos, Haste Intramedular

## Abstract

**Introduction:**

Tibial shaft fracture is the most common long-bone fracture, and the standard treatment is intramedullary (IM) nail fixation. Regardless of the development of this technique pseudoarthrosis remains prevalent.

**Objectives:**

Evaluate the correlation between wedge fragment size and displacement, displacement of the main fragments of the 42B2 type, and pseudoarthrosis incidence.

**Methods:**

We retrospectively assessed all patients with 42B2 type fracture treated with IM nailing between January, 2015 and December, 2019. Six radiographic parameters were defined for preoperative radiographs in the anteroposterior (AP) and lateral views. Another six parameters were defined for postoperative radiographs at three, six, and 12 months. The Radiographic Union Score for Tibial Fractures score was used to assess bone healing.

**Results:**

Of 355 patients with tibial shaft fractures, 51 were included in the study. There were 41 (82.0%) male patients, with a mean age of 36.7 years, 37 (72.5%) had open fractures, and 28 (54.9%) had associated injuries. After statistical analysis, the factors that correlated significantly with nonunion were wedge height > 18 mm, preoperative translational displacement of the fracture in the AP view > 18 mm, and final distance of the wedge in relation to its original anatomical position after IM nailing > 5 mm.

**Conclusion:**

Risk factors for nonunion related to the wedge and42B2 fracture are wedge height > 18 mm, initial translation in the AP view of the fracture > 18 mm, and distance > 5 mm of the wedge from its anatomical position after IM nailing. ***Evidence level III; Retrospective comparative study*** .

## RESUMO

**Introdução:**

A fratura da diáfise da tíbia é a fratura mais comum dentre os ossos longos, sendo o tratamento padrão a fixação com haste intramedular (HIM). Independentemente do desenvolvimento da técnica cirúrgica, a pseudoartrose continua prevalente.

**Objetivo:**

Avaliar a associação entre o tamanho e o desvio da cunha, os desvios dos fragmentos principais do tipo 42B2 e a incidência de pseudoartrose.

**Métodos:**

Avaliamos, retrospectivamente, todos os pacientes com fraturas tipo 42B2 tratados com hastes intramedulares entre janeiro de 2015 e dezembro de 2019. Seis parâmetros radiográficos foram definidos para as radiografias pré-operatórias nas incidências anteroposterior (AP) e perfil. Outros seis parâmetros foram definidos para as radiografias pós-operatórias em 3, 6 e 12 meses de acompanhamento pós-operatório. O Escore Radiográfico de União para as Fraturas da Tíbia (RUST) foi o instrumento usado para avaliar a consolidação óssea.

**Resultados:**

Dos 355 pacientes com fraturas da diáfise da tíbia, 51 foram incluídos no estudo. Os pacientes incluídos foram 41 (82,0%) do sexo masculino, com idade média de 36,7 anos, 37 (72,5%) com fraturas expostas e 28 (54,9%) com lesões associadas. Após análise estatística, os fatores que se correlacionaram significativamente com a não consolidação foram a altura da cunha > 18 mm, o deslocamento translacional pré-operatório da fratura na incidência AP > 18 mm e a distância final da cunha em relação à sua posição anatômica original após a cravação do MI > 5 mm.

**Conclusão:**

Os fatores de risco para a pseudartrose relacionada com a fratura em cunha e42B2 são a altura da cunha > 18 mm, a translação inicial na vista AP da fratura > 18 mm e a distância > 5 mm da cunha em relação à sua posição anatómica após a fixação IM. Nível de evidência III; estudo comparativo retrospectivo. ***Nível de evidência III; Estudo retrospectivo comparativo*** .

## INTRODUCTION

Tibial shaft fractures are the most common fractures of the long bones, accounting for 36.7% of long bone fractures and over 2% of all fractures^1^ . It affects young working-age patients and commonly results from high-energy trauma such as transport accidents (motor vehicle or motorbike) and fall from height^2^ .

For displaced tibial shaft fractures, the most indicated treatment is fixation with an intramedullary (IM) nail because it ensures rapid bone healing and expedites patient's functional recovery.^3 , 4^ Despite this reliable treatment method, a considerable number of patients experience failure during the healing process. The incidence of nonunion after IM nailing varies widely, ranging from 3% to 48%^5^ to a more accepted range of 15–19%.^6 – 8^

Nonunion affects the patient's quality of life by causing physical (pain, disability) and mental hardship^9 , 10^ . There is often a need for secondary intervention or additional treatment to stimulate bone union^11^ . The ability to promptly identify fractures at risk would help to implement preventive strategies to avoid nonunion, improve information given to the patient, and better anticipate the likely healing course ^8 , 12^ .

Some clinical risk factors for nonunion after tibial shaft fracture nailing have been identified in previous studies, such as open fracture, sex, smoking, and fracture of the distal third of the tibia.^13^ There are also some scores to use as predictors for nonunion: Radiographic Union Score for Tibial fractures (RUST), modified RUST, and Non-union Risk Determination score (NURD).^14^ Comminution is considered to be a risk factor ^7^ . To our knowledge, no study has evaluated the influence of the size and displacement of an intact wedge fragment in tibial shaft fractures on the development of nonunion.

The aim of this study was to analyze the influence of wedge fragment size and its preoperative and post-fixation displacement as predictors of nonunion of the third fragment after treatment of AO/OTA 42B2 type fractures treated with IM nailing.

## PATIENTS AND METHODS

This retrospective case series was conducted at an urban university-based level 1 trauma center between January, 2015 and December, 2019. Clinical and radiographic data were collected from patient charts. Written informed consent was obtained from all patients included in this study. Ethical approval was provided by the Scientific and Ethical Committee of the University under protocol 53172921.6.0000.0068 and was performed in accordance with the principles of the Declaration of Helsinki.

The inclusion criteria were as follows: tibial shaft fracture classified as 42B2 according to the AO/OTA classification^15^ treated with IM nailing, age ≥18 years, closed or open Gustilo type I to IIIA,^16^ no previous fracture in the same leg, a minimum of 12 months of follow-up, complete radiographic examination, and signed informed consent.

The exclusion criteria included AO/OTA types A, B3, and C; treatment with anything other than IM nailing; Gustilo type IIIB and IIIC open fractures; contraindication for anesthesia or surgery; infection prior to internal fixation; articular extension of the fracture; pathologic fracture; stress fractures; age <17 years; and follow-up <12 months.

Baseline demographic data on the following were collected: age, sex, associated injuries, AO/OTA classification, and Gustilo classification of open fractures. Infection was defined according to the criteria for fracture-related infection.^17^ .

Radiographs used to measure the fracture parameters were the preoperative radiograph and 3-, 6-, and 12-month post operative radiographs.

**Figure 1:**
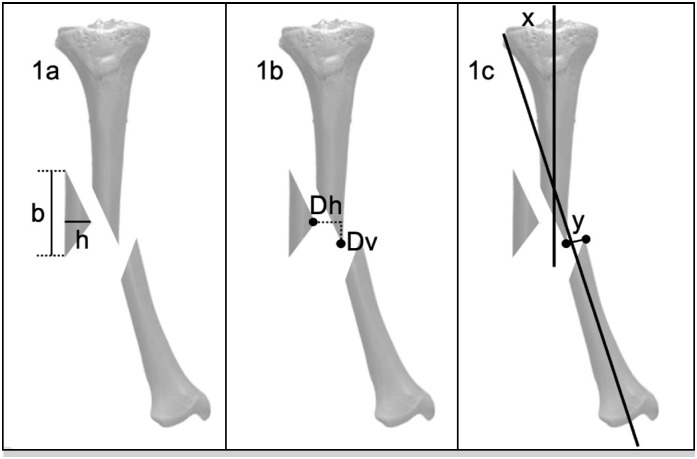
Radiographic measurements of the wedge size and displacement and the initial fracture displacement.

In the preoperative radiograph, the size of the wedge fragment was measured as follows: b = length of the cortical bone measured at the base of the wedge and h = height of the wedge measured with a line perpendicular to the base to the apex of the wedge( Figure 1a ). The displacement of the wedge fragment was defined as the vertical distance from its original position in the proximal fragment (Dv) and the horizontal distance (Dh) from the apex of the wedge fragment ( Figure 1b ). Fracture displacement (x) was measured as the angle between the anatomical axis of the proximal fragment and anatomical axis of the distal fragment; and fracture translation (y) was measured as the distance between the most distal point of the proximal main fragment and most proximal point of the main distal fragment ( Figure 1c ).

**Figure 2:**
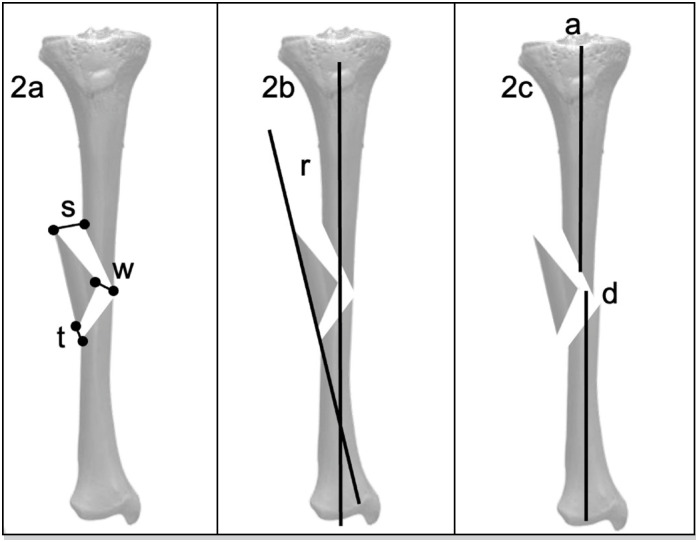
Post operative radiographic measurements of the wedge and the fracture displacement.

Three months postoperatively, the reduction of the wedge fragment was measured as the distance between the proximal (s), apex (w), and distal (t) points to its original position in reduced and fixed fractures ( Figure 2a ). Angulation of the wedge fragment (r) was measured as the angle between the line parallel to the cortical bone of the base of the wedge and the anatomical axis of the tibia ( Figure 2b ). Reduction of the anatomical axis of the tibia (x) and the final gap “y” in the fracture site were also measured ( Figure 1c ).

Radiographic fracture healing was evaluated using the RUST, which assigns points based on the assessment of healing visible in anteroposterior (AP) and lateral (L) radiographs, with 1 point assigned if there is a fracture line with no callus, 2 points if there is callus present but a fracture line is still visible, and 3 points if there is a bridging callus with no evidence of a fracture line. Individual cortical scores are added to obtain a total score. A minimum of 9 points is used to exclude nonunion and 12 points to consider the fracture definitively healed.^18^ This was done using the radiographs obtained at 6 and 12 months.

The healing status of the wedge to the tibial main fragments was also recorded if the wedge was completely healed on both sides, or only proximal or distal.

All radiographic measurements and assessments of healing (RUST) were performed independently by three authors. For the measurements, the mean was accepted for the analysis, and for the RUST, a consensus was reached after the first evaluation.

Statistical analyses were performed using SigmaPlot software (version 11.0; SPSS, Richmond, CA, USA). Descriptive statistics included means and standard deviations for continuous variables and counts (percentages) for categorical variables. The correlation between the aforementioned radiographic parameters and RUST was analyzed using a linear regression test. Similarly, the correlation with wedge fragment healing was analyzed using the Pearson and Hosmer-Lemeshow tests. Statistical significance was set at 0.05.

## RESULTS

Between January, 2015 and December, 2019, 355 patients were diagnosed with tibial shaft fracture. According to the AO/OTA classification, 51 fractures (14%) were classified as type B2 (presence of an intact wedge fragment) and fulfilled the inclusion criteria. Of these patients, 41 (82%) were male and 10 (18%) were female, with a mean age of 36.7 years (range, 17–70) years. The left side was fractured in 31 (60.8%) patients. The fracture was open in 37 (72.5%) patients and associated injuries were present in 28 (54.9%) patients. During the follow-up, five (9.8%) patients developed infection ( Table 1 ).

**Table 1:** Demographic characteristics of the patients.

Variable	Description (n = 51)
**Age (years)**	
Mean ± SD	36.7 ± 13.6
**Gender, n (%)**	
Female	10 (19.6)
Male	41 (80.4)
**Side, n (%)**	
Right	20 (39.2)
Left	31 (60.8)
**Associated injuries, n (%)**	
No	23 (45.1)
Yes	28 (54.9)
**Open fractures, n (%)**	
No	14 (27.4)
Yes	37 (72.6)
**Post op infection, n (%)**	
No	46 (90.2)
Yes	5 (9.8)

The measurements of the wedge were a mean height (h) in the AP view of 18.6 ± 7.4 mm and in the L view 19.9 ± 9.1 mm. The base length (b) in the AP view was 57.4 ± 25.3 mm and in the L view 54.7 ± 26.9 mm.

Regarding the initial displacement of the wedge from its original position in the AP view was a horizontal distance (Dh) of 11.3 ± 10.4 mm and a vertical distance (Dv) of 12.8 ± 14.9 mm. In the L view Dh was 9.7 ± 9.6 mm, and Dv was 10.5 ± 11.3 mm.

In the AP view, the fracture's initial displacement measured by the mechanical axis (x) was 8.0° ± 6.8 in the AP view and 6.0° ± 4.9 in the L view. The translational displacement (y) was 18.3 ± 11.9 mm in the AP view and 15.7 ± 10.8 in the L view.

The displacement of the wedge from its original position after reduction and IM nailing of the tibia were measured three times. The distance from the apex of the wedge to the proximal fragment (w) in the AP view was 4.9 ± 7.2 mm and 4.6 ± 3.7 mm in the L view. The distance of the most proximal point to the proximal fragment (s) in the AP view was 4.8 ± 4.3 mm and 6.4 ± 4.7 mm in the L view. The distance of the most distal point to the distal fragment (t) was 5.4 ± 4.9 mm in the AP view and 6.5 ± 6.9 mm in the L view.

The angulation of the wedge to the mechanical axis (r) was 6.0° ± 6.8 in the AP view and 5.3° ± 4.9 in the L view.

The final tibial mechanical axis alignment (a) was 1.5° ± 1.5 in the AP view and 2.4° ± 2.7 in the L view. The final gap at the fracture site (d) was 0.9 ± 1.5 mm in the AP view and 2.0 ± 3.0 mm in the L view.

Results of the radiographic measurements are presented in the Table 2 .

**Table 2:** Statistical analysis of the radiographic parameters of the wedge size and displacement and fracture displacement pre and post operative in the AP and L view.

Parameters	Mean and SD (mm)	Correlation with RUST 12 month		Correlation with healing 12 month	
		R	p	OR [I.C. 95%]	p
b (AP)	57,4 (25,3)	0,050	0,232	0,991 [0,965-1,018]	0,501
b (L)	54,7 (26,9)	0,029	0,423	0,995 [0,967-1,024]	0,738
h (AP)	18,6 (7,4)	0,144	0,039	0,888 [0,781-1,009]	0,068 *
h (L)	19,9 (9,1)	0,245	0,014	0,875 [0,727-0,964]	0,042 *
Dv (AP)	12,8 (14,9)	0,078	0,135	0,969 [0,918-1,023]	0,252
Dv (L)	10,5 (11,3)	0,081	0,175	0,948 [0,872-1,031]	0,214
Dh (AP)	11,3 (10,4)	0,071	0,156	0,994 [0,931-1,061]	0,852
Dh (L)	9,7 (9,6)	0,051	0,291	0,937 [0,850-1,033]	0,191
x (AP)	8,0 (6,8)	0,000	0,918	1,000 [0,909-1,101]	0,998
x (L)	6,0 (4,9)	0,024	0,473	1,068 [0,914-1249]	0,406
y (AP)	18,3 (11,9)	0,157	0,016	0,929 [0,865-0,997]	0,042 *
y (L)	15,7 (10,8)	0,041	0,342	0,984 [0,886-1,092]	0,761
s (AP)	4,8 (4,3)	0,022	0,483	1,002 [,825-1,216]	0,985
s (L)	6,4 (4,7)	0,00	0,741	1,017 [0,871-1,186]	0,833
w (AP)	4,9 (7,2)	0,000	0,904	1,066 [0,773-1,471]	0,695
w (L)	4,6 (3,7)	0,003	0,815	1,064 [0,828-1,368]	0,626
t (AP)	5,4 (4,9)	0,223	0,020	0,815 [0,658-0,941]	0,032 *
t (L)	6,5 (6,9)	0,006	0,689	1,023 [0,926-1,143]	0,595
r (AP)	6,0 (6,8)	0,099	0,118	1,215 [0,987-1,494]	0,066
r (L)	5,3 (4,9)	0,001	0,903	1,178 [0,963-1,440]	0,111
a (AP)	1,5 (1,5)	0,004	0,744	1,416 [0,844-2,377]	0,188
a (L)	2,4 (2,7)	0,009	0,611	1,299 [0,918-1,840]	0,140
d (AP)	0,9 (1,5)	0,036	0,307	0,630 [0,299-1,325]	0,223
d (L)	2,0 (3,0)	0,008	0,618	0,949 [0,739-1,219]	0,684

SD: standard deviation; AP; anteroposterior view; L: lateral view; b: base length of the wedge; h: height of the wedge; Dh: horizontal distance wedge-original location; Dv: vertical distance wedge-original location; x: preop mechanical axis displacement; y: preop translational displacement; s: distance between the most proximal point of the wedge to its anatomical position after fixation; t: distance between the most distal point of the wedge to its anatomical position after fixation; w: distance between the apex of the wedge to its anatomical position after fixation; r: angle between the wedge and the mechanical axis after fixation; a: mechanical axis after fixation and d: gap at the fracture site after fixation. Correlation with RUST: linear regression test; correlation with healing: Pearson and Hosmer-Lemeshow test.

* statistically significant.

The mean RUST at the 6-month follow-up was 8.1 ± 1.6 (range, 5–11), with 24 (47.1%) fractures with a score higher than 9 and none with a score of 12. At the 12-month follow-up, the mean RUST was 10.1 ± 1.5 (range, 6–12), with a total of nine (17.6%) patients with a score of 12, and 32 (62.7%) with a score of 9 to 11. At the completion of the follow-up, 10 (19.7%) patients were considered to have a nonunion with RUST between 6 and 8.

In the analysis of the wedge union in the 10 patients with nonunion, five (50%) fractures presented with nonunion of the wedge both proximally and distally, one (10%) showed healing only in the proximal part of the wedge, and four (40%) showed healing only in the distal part of the wedge.

The statistical analyses are presented in Table 2 . After the linear regression test to find out the correlation of the measurements and RUST and the Pearson Hosmer-Lemeshow test for the correlation with healing of the wedge both at 12-month follow-up, we could find correlation with only three parameters: “h” height of the wedge fragment in the both AP and L view (OR = 1.183 [1.014-1.422] / p = 0.048), ‘y” preoperative translational displacement in the AP view (OR = 1.111 [1.013-1.218] / p = 0.025) and “t” distance of the most distal point of the wedge in post operative radiographs (OR 1.311 [1.126-1.504] / p = 0.004) ). The impact of these three parameters on the RUST ranged from 14% to 24%, as depicted in Table 2 .

## DISCUSSION

The tibial shaft is the most frequently fractured long bone,^19^ and despite the introduction of minimally invasive intramedullary nail fixation, complications remain prevalent.^11^ Nonunion can be a devastating complication to patients and a burden to the public health system^9 , 20^ , with an incidence varying from 15% to 19%.^7^

The ability to predict fractures that develop nonunion could allow surgeons to anticipate the problem and institute prevention strategies in the early management, define an appropriate surveillance during follow-up and early intervention to promote healing, and ultimately decrease both the patient suffering and cost to the health system ^3 , 6 , 8^ .

Several studies have been conducted to identify risk factors for nonunion in tibial shaft fractures fixed with IM nails. These studies found factors related to the patient: ASA physical status score, Injury Severity Score, smoking status, comorbidities, and gender ^3 , 5 , 8 , 21^ . Factors related to the fracture included open injuries, high energy, comminution, AO/OTA type B or C, fibular fracture, and associated injuries.^7 , 8 , 22^ Recently, more focus has been placed on fracture gap as a high-risk factor^23 , 24^ .

Some scores were also developed to assess healing evolution and predict nonunion, such as the RUST, modified RUST, and NURD.^14^

As cited above, comminuted fracture is a risk factor for nonunion, and AO/OTA types B and C are considered comminuted but have different characteristics. On the one hand, type B has less soft tissue injury than type C, but the wedge fragment in type B can be totally avascular.

The incidence of AO/OTA type B fractures is considerable and varies between 22% and 40%^25 , 26^ ; therefore, it is worth evaluating wedge size and displacement in the development of nonunion as risk factors. We decided to include only type B2 fractures because they had an intact wedge. Type B3, with a fragmented wedge, may behave as a type C fracture. As we could not find any study similar to this, we defined radiographic measurements as shown in figures 1 and 2 to understand the most relevant measurements that could lead to nonunion.

Our findings in the 3-month radiograph with very few patients showing signs of bone healing corroborate the findings of other authors, such as Mundi et al.^27^ and Wojahn et al.,^28^ who found that the median time to radiographic union after tibial nailing was approximately 20 weeks, and little healing occurred within the first 8 weeks after surgery.

The RUST objectively determines the extent of healing by scoring the degree of fracture healing from 1 to 3 points for each of the four cortices, as viewed from AP and L radiographs. The sum of 12 points is a completely healed fracture in the four cortices, and points 9–11 indicate bone healing in three cortices, which can be considered a good result.^18^

At the 6-month follow-up, close to half of the patients (47.1%) had RUST higher than 9 points and could have been considered to be healed. By 12 months, the number had increased to 80.3%, with a score higher than 9 points. This is an indication that the healing of a 42B2 fracture can take between 6 and 9 months, and not performing surgery for nonunion in all fractures not healed within 6 months may be a wise decision.

In our series, 10 (19.7%) patients were diagnosed with nonunion after 12 months. The statistical analysis of the correlation between radiographic measurements and nonunion revealed positive correlation with three parameters: “h,” height of the wedge; “y,” preoperative translational displacement; and “t,” post operative distance of the wedge to its anatomical position.

These results indicate that the height of the wedge is more important than its length. This may be because a wedge with a larger height compromises the diameter of the tibial shaft, leaving less contact area between the two main fragments of the tibia. This is consistent with the finding that less bone contact leads to nonunion^7,24.^

Many articles cite high energy trauma as a risk factor without being more specific. Our results showed that the risk factor was the initial translational displacement between the main proximal and distal fragments of the tibia in the AP view. Translational displacements > 18 mm in the AP view may be considered a risk factor for nonunion. This may be related to soft tissue injury and vascular compromise of the fracture site, worsening, and delaying the healing process.

Another risk factor was the final distance of the wedge to its original anatomical position, which was measured in this study as the distance between the apex of the wedge and original position (t). An average distance of 5 mm showed a positive correlation with the development of nonunion.

Our study has several limitations. The first limitation is its retrospective design. The second limitation was the small number of patients, which influenced the statistical analysis. Any radiographic measurement may be inconsistent because of the magnification of the image and imprecise measurements. Even if the RUST is only 3 points for each cortex, it is a subjective assessment and is capable of being erroneous. The lack of analysis of some variables may also interfere with the results.

In conclusion, in AO/OTA 42B2, risk factors for nonunion are size of the wedge, especially its height (> 18 mm); initial translation of the fracture (> 18 mm); and final reduction of the wedge fragment (> 5 mm). In the presence of these factors, one can consider either initiating a different strategy or not waiting long to perform surgery to ensure bone healing.

## CONCLUSIONS

The risk factors identified in this study for nonunion in 42B2 tibial shaft fractures treated with IM nailing are as follows:

Wedge height > 18 mm

Translational displacement on AP preoperative radiograph > 18 mm

Distance of the wedge from its original position on postoperative radiograph > 5 mm
